# Regulation of T Cell Responses by Nano-Hydroxyapatite to Mediate the Osteogenesis

**DOI:** 10.3389/fbioe.2022.884291

**Published:** 2022-04-04

**Authors:** Fangze Guo, Changqing Yuan, Hailin Huang, Xuyang Deng, Zirui Bian, Danyang Wang, Keke Dou, Li Mei, Qihui Zhou

**Affiliations:** ^1^ Department of Stomatology, The Affiliated Hospital of Qingdao University, Qingdao University, Qingdao, China; ^2^ School of Stomatology, Qingdao University, Qingdao, China; ^3^ Institute for Translational Medicine, The Affiliated Hospital of Qingdao University, Qingdao University, Qingdao, China

**Keywords:** nano-hydroxyapatite, T cell, osteogenesis, osteoimmunology, nanomaterials

## Abstract

Nano-hydroxyapatite (nHA) has been widely applied as a tissue-engineering biomaterial and interacted with osteoblasts/stem cells to repair bone defects. In addition, T cells that coexist with osteoblasts/stem cells in the bone modulate the regulation of osteoimmunology by cytokine formation. However, the effects of nHA on T cells and the following regulatory interplay on osteogenic differentiation have been rarely examined. In this work, the physicochemical properties of needle-like nHA are characterized by field emission scanning electron microscopy, zeta potential, Fourier transform-infrared and X-ray diffraction. It is found that as the concentration of nHA increases, the proliferation of T cells gradually increases, and the proportion of apoptotic T cells decreases. The percentage of CD4^+^ T cells is higher than that of CD8^+^ T cells under the regulation of needle-like nHA. Furthermore, the supernatant of T cells co-cultured with nHA significantly inhibits the osteogenic differentiation of MC3T3-E1 by downregulating the formation of alkaline phosphatase and calcium nodule compared with the supernatant of nHA. Thus, our findings provide new insight into the nHA-mediated T cell and osteoblast interactions.

## 1 Introduction

Hydroxyapatite (HA) is one of the most remarkable biomaterials and has been widely used in bone tissue engineering and regenerative medicine owing to its good biocompatibility and osteoinductive activity (Wei and Ma, 2004; Santos et al., 2012; Wang et al., 2012; Akram et al., 2014; Pepla, 2014; Yu et al., 2022). Particularly, nano-HA (nHA) particles gained increasing attention from the viewpoint of fundamental research and clinical applications due to their enhanced (bio)physicochemical properties compared with HA, including solubility, surface energy and bioactivity (Wang et al., 2004, 2021a; Guo et al., 2007; Mokabber et al., 2019; Ji et al., 2021). In addition, nHA is more similar to apatite of biological bone and dental tissues in terms of morphology, crystal structure and crystallinity (Fathi et al., 2008; Barros et al., 2019). It has been demonstrated that nHA can greatly promote osteoblast proliferation and osteogenesis, as well as accelerate osseointegration (Wang et al., 2021a). In addition, T cells that coexist with osteoblasts in the bone modulate the regulation of osteoimmunology by cytokine formation. Osteoimmunology is an emerging field for investigating the interaction between the immune system and bone tissue (Kumar and Roger, 2019; Yu et al., 2022). Osteoblasts have the capacity to communicate with the immune cells and vice versa (Gruber, 2019; Tsukasaki and Takayanagi, 2019; Zhang et al., 2020). Therefore, as a key regulator in the process of bone regeneration, it is vital to elicit how nHA mediates the interplay between osteoblasts and T cells.

The bone marrow is the major location of haematopoiesis, harboring haematopoietic stem cells, myeloid and lymphoid progenitors, and mature immune cells such as B cells, neutrophils, macrophages, and T lymphocytes (Tsukasaki and Takayanagi, 2019). Osteoblasts/stem cells and immune cells share the same microenvironment and interact to complete the function of the “bone immune system” (Kumar and Roger, 2019). Osteoblast maturation is triggered by T lymphocytes (Croes et al., 2016; Khassawna et al., 2017). The formation and activity of osteoclasts are affected by some cytokines and growth factors secreted by T lymphocytes (Gillespie, 2007). Activated conventional CD4^+^ and CD8^+^ T lymphocytes control bone homeostasis *via* surface-bound molecules that bind to cognate molecules expressed in osteoblasts and their stromal cell precursors and by producing osteoclastogenic cytokines such as receptor activator of nuclear factor-κB ligand, tumor necrosis factor α (TNF-α), interleukin 1 (IL-1), IL-6, and IL-17 (Pacifici, 2016). In addition, T cell subsets and their released factors may influence bone regeneration outcomes differentially. CD4^+^ and CD8^+^ T lymphocytes affect osteoprogenitor cell development in various ways (Kumar and Roger, 2019; Shao et al., 2020). Nanoparticles are known to be able to interact with and affect the immune system (Liu et al., 2009). Nanoparticles could induce T cell activation and differentiation (Items et al., 2018). The effect of nHA with different aspect ratios on T cells has been investigated and greatly affected CD3^+^ T cells as percentages of total cells (Yu et al., 2022). However, the effects of various nHA concentrations on T cells and the following regulatory interplay on osteogenic differentiation have been rarely examined.

**FIGURE 1:**
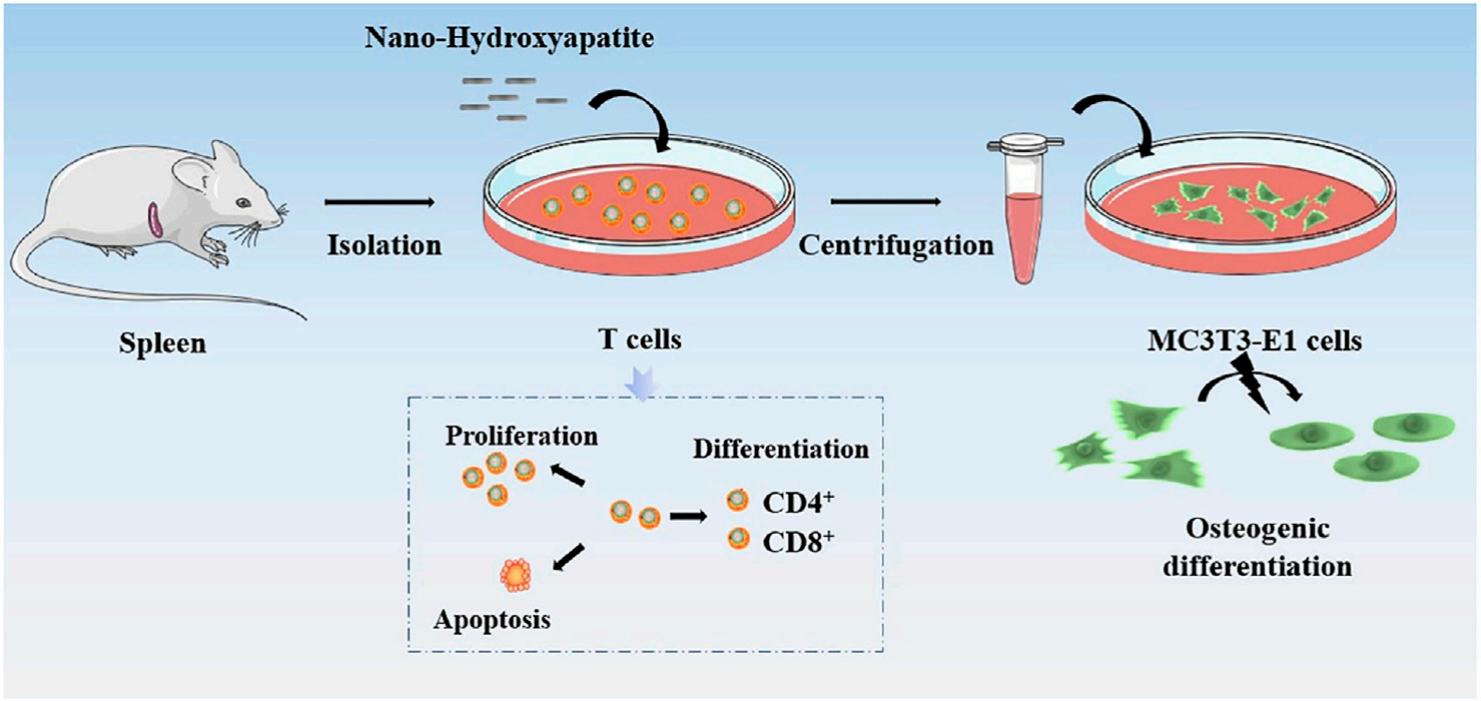
Schematic diagram of the effects of nHA on T cell responses, differentiation, and the following regulatory on osteoblasts.

Inspired by the introduction above, nHA was applied to investigate their effects on T cell responses, differentiation, and the following regulatory on osteoblasts (Figure 1). First, the physicochemical properties of needle-like nHA are characterized by field emission scanning electron microscopy (FE-SEM), zeta potential, Fourier transform-infrared (FT-IR) and X-ray diffraction (XRD). The viability, apoptosis and differentiation of T cells were treated with different nHA concentrations (i.e., 0, 10, 100, and 1,000 μg/ml), and cell viability was determined. In addition, the supernatant after co-culturing nHA and T cells was used to test its effect on the osteogenic capacity of osteoblasts by measuring the formation of alkaline phosphatase and calcium nodule. Thus, this work could offer new insight into the nHA-mediated T cell and osteoblast interactions.

## 2 Materials and Methods

### 2.1 Materials

The nHA (purity >97%, CAS 1306-06-5) was purchased from Aladdin Industrial Inc. (Shanghai, China). Cell Counting Kit-8 (CCK-8), Annexin V-FITC/PI apoptosis detection kit, bicinchoninic acid (BCA) protein assay kit, Dulbecco’s modified Eagle’s medium (DMEM), trypsin, fetal bovine serum (FBS), penicillin and streptomycin were supplied by Meilunbio (Dalian, China). EasySep™ mouse T cell isolation kit was obtained from StemCell Technologies (Vancouver, CA, United States). Anti-mouse CD3 (Clone 17A2), anti-mouse CD28 (Clone 37.51) were purchased from Peprotech (Westlake Village, CA, United States). Anti-mouse CD4, phycoerythrin (PE, Clone GK1.5), Anti-mouse CD8α, phycoerythrin-cyanine 7 (PE-Cy7, Clone 53-6.7) and flow cytometry staining buffer was purchased from Multi Sciences Biotech, Co., Ltd. (Hangzhou, China). 5-(and-6)-Carboxyfluorescein diacetate, succinimidyl ester (CFSE) was obtained from Abbkine Bioadvisers. Alizarin red S (ARS) staining kit for osteogenesis was purchased from Beyotime (Shanghai, China). Dimethyl sulfoxide (DMSO) and RPMI 1640 culture medium were obtained from Solarbio (Beijing, China). Deionized (DI) water was used in all experiments. All chemicals were used without additional purification.

### 2.2 Physicochemical Characterization of nHA

#### 2.2.1 FE-SEM

The size and morphology of nHA were evaluated using an FE-SEM (Sigma500, Zeiss, Germany). The nanoparticles were diluted in DI water and placed in an ultrasonic bath for 30 min to disperse the particles. The entire observation procedure was carried out in a vacuum atmosphere with a constant accelerating voltage of 5 kV, at magnification of 50,000. The average crystal size was determined by ImageJ software. The size was determined statistically by averaging data from 100 unique particles from different images.

#### 2.2.2 FT-IR Spectroscopy

To measure the FT-IR spectra of the nHA, a Nicolet iN10 FT-IR spectrometer (Thermo Fisher Scientific, Waltham, MA, United States) was utilized over the range of 500–4,000 cm^−1^ at a scanning resolution of 2 cm^−1^ during 32 scans.

#### 2.2.3 XRD Spectroscopy

The crystal structures of nHA were measured using XRD spectroscopy (Ultima IV Japan). The nHA was examined between 20° and 60° (2θ) at a scanning rate of 0.02° (2θ) per min operating with voltage 40 kV and current 40 mA equipped with Cu Kα radiation (λ = 1.5418 Å).

#### 2.2.4 Zeta Potential

Dynamic light scattering on a Zetasizer Nano ZSE (United Kingdom) was used to assess the zeta potential of nHA samples. At the same concentration, the nHA were disseminated in DI water and 1640 RPMI complete medium.

### 2.3 Cell Assays

#### 2.3.1 Isolation and Culture of Mouse T Cells

Spleens were dissected from C57BL/6 mice. Following mechanical digestion, the tissue suspension was filtered to produce a leukocyte single-cell suspension. T cells were isolated from this suspension by a mouse T cell isolation kit (19851; StemCell). These separated T lymphocytes were then used in the following cellular assays.

T cells (10^5^ cells/ml) were cultured in 1640 RPMI complete medium (with FBS) in the presence of anti-mouse CD3 and anti-mouse CD28 antibodies. T cells co-cultured in the presence of 10, 100, and 1,000 µg/ml nHA. T cells were cultivated under the same conditions without the addition of nHA as the control.

The Cell Culture Centre of Shanghai Institutes for Biological Science Chinese Academy of Sciences (Shanghai, China) contributed a murine osteoblastic cell line (MC3T3-E1). MC3T3-E1 cells were incubated at 37°C in 5% CO_2_ and the medium was changed every 2 days. Cells were subcultured when they achieved 80%–90% confluence by using Dulbecco’s phosphate buffered saline and trypsin/ethylenediaminetetraacetic acid. The studies listed below were carried out in duplicate across three different experiments (*n* = 3). This research was approved by the Ethics Committee of the Affiliated Hospital of Qingdao University (approval number: QYFYWZLL 26848).

#### 2.3.2 T Cell Viability

The CCK-8 was used to assess cellular viability. Briefly, nHA samples were added into T cells medium (10^5^ cells/well in 96-well plates) for 1, 2 and 3 days. Subsequently, 10 μl of CCK-8 solution was added to each well. The option density (OD_450_) was measured using a microplate reader (SynergyH1/H1M, Bio-Tek, China) after 4 h of incubation.

#### 2.3.3 Flow Cytometry

T cell apoptosis was examined by using the Annexin V-FITC/PI Apoptosis Assay Kit according to the manufacturer’s instructions. Briefly, T cells were incubated with or without nHA samples for 48 h. T cells were collected, washed with phosphate buffer solution (PBS), and resuspended at a concentration of 10^6^ cells/ml in 1 × binding buffer. Then 100 μl of the solution (10^5^ cells) was moved to a 2.5 ml Eppendorf tube, and 5 μl of annexin V-fluorescein-5-isothiocyanate (FITC) and 5 μl of propidium iodide (PI) were added. T cells were gently vortexed and incubated in the dark for 15 min. All samples were analyzed using FACScan flow cytometry (ACCURI C6, Becton Dickinson, United States). 10,000 ungated events were collected for each sample. The early apoptotic populations were represented by Annexin V^+^ PI^−^ cells. The late apoptotic or secondary necrotic populations were represented by Annexin V^+^ PI^+^ cells.

T cells were stained with PE-conjugated anti-mouse CD4 and PE-cy7 conjugated anti-mouse CD8 antibodies after 5 days of incubation. T cells were washed and resuspended in flow cytometry staining buffer. FACScan flow cytometry was used to analyze the samples.

T lymphocytes were isolated, stimulated with anti-mouse CD3 and anti-mouse CD28 and labeled with 2.5 μM CFSE. These cells were cultured in the presence of nHA in 96-well plates at an equal ratio. After 72 h, the proliferation was assessed using flow cytometry based on CFSE dilution in gated T cells. Flow Cytometry samples were analyzed using FlowJo software.

#### 2.3.4 MC3T3-E1 Cell Viability

DMEM (CON), the supernatant of nHA in 1640 RPMI complete medium (nHA), the supernatant of T cells (T), T cells and 100 μg/ml nHA co-culture supernatant (T + nHA) were respectively added into MC3T3-E1 cells medium (5 × 10^3^ cells/well in 96-well plates) for 1 and 3 days culture. Then 10 μl CCK-8 solution was added to each well. The OD_450_ was measured using a microplate reader after 1 h of incubation.

#### 2.3.5 Alkaline Phosphatase Activity

ALP activity was used to estimate the osteogenic performance of T cells and 100 μg/ml nHA co-culture supernatant, for evaluating the early osteogenic differentiation of MC3T3-E1 cells. MC3T3-E1 cells (2 × 10^4^ cells/well in 24-well plates) were seeded on the samples and incubated with the osteogenic induction medium. The osteogenic induction medium (CON), the supernatant of nHA in 1640 RPMI complete medium (nHA), the supernatant of T cells (T), T cells and 100 μg/ml nHA co-culture supernatant (T + nHA) were respectively added into MC3T3-E1 cells medium. Following a 14 days culture period, the ALP activity of MC3T3-E1 cells was determined using an ALP test kit and the manufacturer’s instruction. A BCA protein assay kit was used to standardize the total protein content of cells (king unit/g prot), which was used to calculate ALP activity.

### 2.4 ARS Staining

To examine matrix mineralization qualitatively, at the differentiation days of 14, MC3T3-E1 cells were washed with PBS, and then fixed and stained with ARS Staining Kit for Osteogenesis (C0148, Beyotime) as the manufacturer’s instructions. The cells were examined by light microscopy (VIYEE, China).

### 2.5 Statistical Analysis

All data were presented as mean ± standard deviation (SD). GraphPad Prism 8.0 or Origin 9.0 software was used for statistical analysis. To determine differences between groups, one-way analysis of variance (ANOVA) with Tukey’s test was used. A value of *p* < 0.05 was considered to be statistically significant.

## 3 Results and Discussion

### 3.1 Physicochemical Characterization of nHA

**FIGURE 2:**
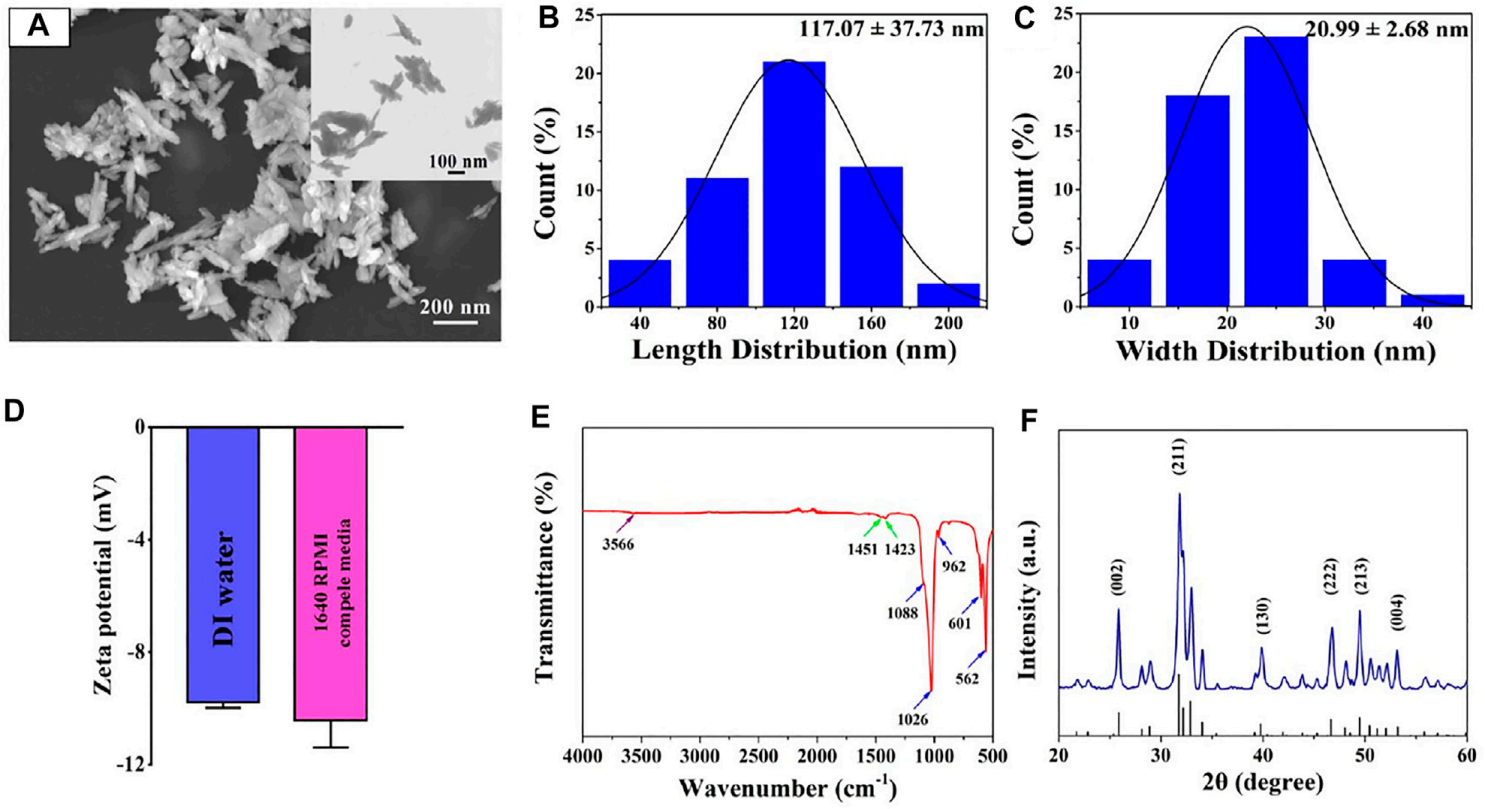
**(A)** FE-SEM image of nHA. **(B,C)** Length and width size distribution of nHA. **(D)** Zeta potential of nHA in DI water and 1640 RPMI complete medium. **(E)** FT-IR spectrum of nHA. The purple, green and blue arrows indicated OH-, CO32- and PO43- groups, respectively. **(F)** XRD pattern of nHA.

Accumulating evidence suggested that the morphology and size of nanomaterials play a critical role in cell behaviors, including adhesion, viability, proliferation, migration, functionalization or differentiation (Nguyen et al., 2016; Zhou et al., 2018; Li et al., 2020; Liu M. et al., 2021; Wang et al., 2021b; Yang et al., 2021; Yao et al., 2021). Figure 2A shows that the nanosized HA particles possessed uniform distribution and needle-shaped morphology. Figures 2B,C displays that the length of the needle-like HA nanoparticle was 117.07 ± 37.73 nm and their width was 20.99 ± 2.68 nm. Previous studies have demonstrated that the needle-shaped nHA were biocompatible with bone marrow mesenchymal stem cells, enhanced cell proliferation and facilitated osteogenic differentiation (Paquin et al., 2015). Also, it was reported that the size (20–40 nm) of apatite nanoparticles could promote biomineralization in the bone tissue (Cai et al., 2007).

In addition, the surface charge and functional group in the nanomaterials significantly affected their interplay with cells (Thian et al., 2010). As shown in Figure 2D, it was found that the zeta potential of nHA in the DI water is −9.78 ± 0.2 mV. The nHA with negative charges was consistent with results reported in other studies (Huang et al., 2014). When nanoparticles were suspended in the cell culture medium, additional proteins were attached to their surface, which could affect their zeta potential (Thian et al., 2010; Sabuncu et al., 2012; Patel et al., 2017). In the 1640 RPMI complete medium, the zeta potential of the nHA was −10.43 ± 0.97 mV. The result indicates that nHA in the 1640 RPMI complete medium had no effect on their surface charge.

Moreover, FT-IR spectra were acquired to detect the functional group of the nHA sample. As indicated in Figure 2E, the spectroscopy of nHA displayed characteristic bands, including PO_4_^3-^ (1,088, 1,026, 962, 601 and 562 cm^−1^) and OH^-^ (3,566 cm^−1^). In addition, the ion stretching vibration at 1,451 and 1,423 cm^−1^ confirmed the presence of the CO_3_^2-^ group. The functional groups of nHA from FT-IR spectra analysis are compared with the results in other studies (Cipreste et al., 2016). The XRD pattern of nHA showed several sharp peaks, indicating good crystallinity (Figure 2F). The characteristic XRD peaks (25.8°, 31.8°, 39.8°, 46.7°, 49.5°, and 53.2°) corresponded to the (002), (211), (130), (222), (213) and (004) crystal planes of the nHA structure, respectively. The peak positions are in good agreement with the ICSD card no. 74-0566.

### 3.2 The Interactions Between nHA and T Lymphocytes

**FIGURE 3:**
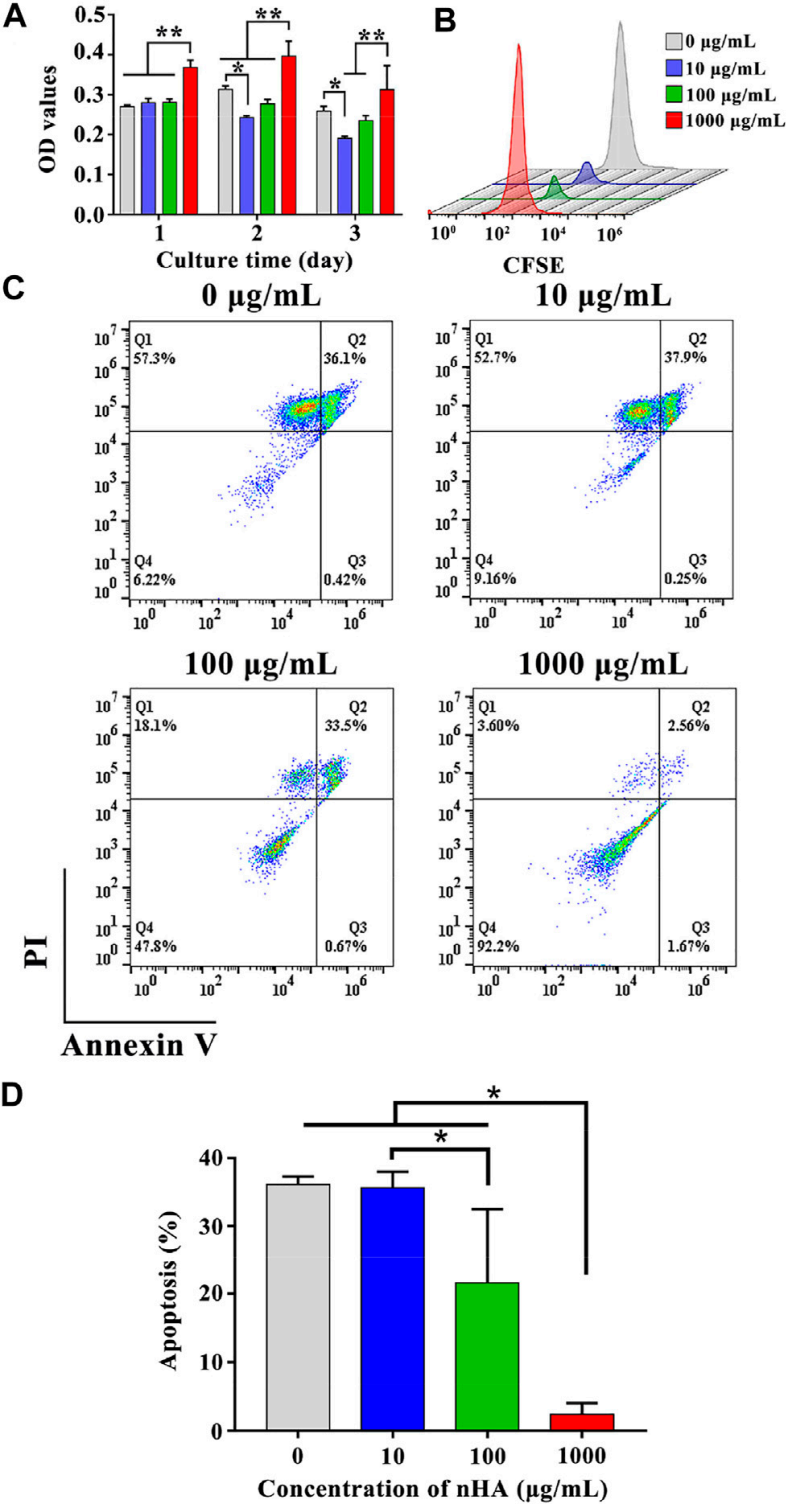
**(A)** T cells viability on various samples for 1, 2 and 3 days. * represents *p* < 0.05 and ** represents *p* < 0.01. **(B)** Effect of nHA on T cell proliferation. **(C)** Flow cytometry analysis of the effect of nHA on T cell apoptosis. **(D)** Quantitative analysis of apoptosis.

T cells were co-cultured with various concentrations of nHA to assess the effect of nHA on T cell viability. After 1, 2, and 3 days of cell culture, CCK-8 viability assays were performed. After 1 day, the cell viability in 1,000 μg/ml nHA was greatly higher than those in other groups, as shown in Figure 3A. There were no significant differences among control, 10 and 100 µg/ml nHA. After 2 and 3 days, the cell viability in 10 μg/ml nHA was lower than that in the control group. However, in the 100 μg/ml nHA group, the cell viability was similar to the control group. The cell viability in the 1,000 μg/ml nHA group was higher than 10 and 100 µg/ml groups. In addition, as the incubation time increased from 1 to 3 days, the cell viability in 10 μg/ml nHA significantly decreased and the cell viability of the 100 μg/ml nHA group remained unchanged. These results imply that the viability of T cells was dependent on the concentrations of nHA. Importantly, 1,000 μg/ml nHA greatly enhanced the viability of T cells. We added different concentrations of nHA to the cell culture medium to explore the influence of nHA content on T cell proliferation after 3 days. As shown in Figure 3B, there was only one CFSE signal peak in each group, which represented the same number of generations of cell proliferation in each group. However, the CFSE signal of the 1,000 μg/ml nHA group was the strongest, indicating that the number of cells in the sample was the most among other groups, which was consistent with the result of T cell CCK-8. Furthermore, the effect of nHA on the apoptosis of T cells with different concentrations of nHA was shown in Figure 3C. It was found that the percentages of apoptotic cells in control, 10 and 100 µg/ml groups were 36.1%, 37.9% and 33.5%, respectively. The percentage of apoptosis cells in the 1,000 μg/ml nHA group (2.56%) was the lowest than those in other groups. The percentages of viable cells in control, 10, 100 and 1,000 µg/ml groups were 6.22%, 9.16%, 47.8% and 92.2%, respectively. Taken together, as the concentration of nHA increased, the proportion of apoptotic T cells gradually decreased after 48 h culture, and the proportion of normal living cells gradually increased (Figure 3D).

**FIGURE 4:**
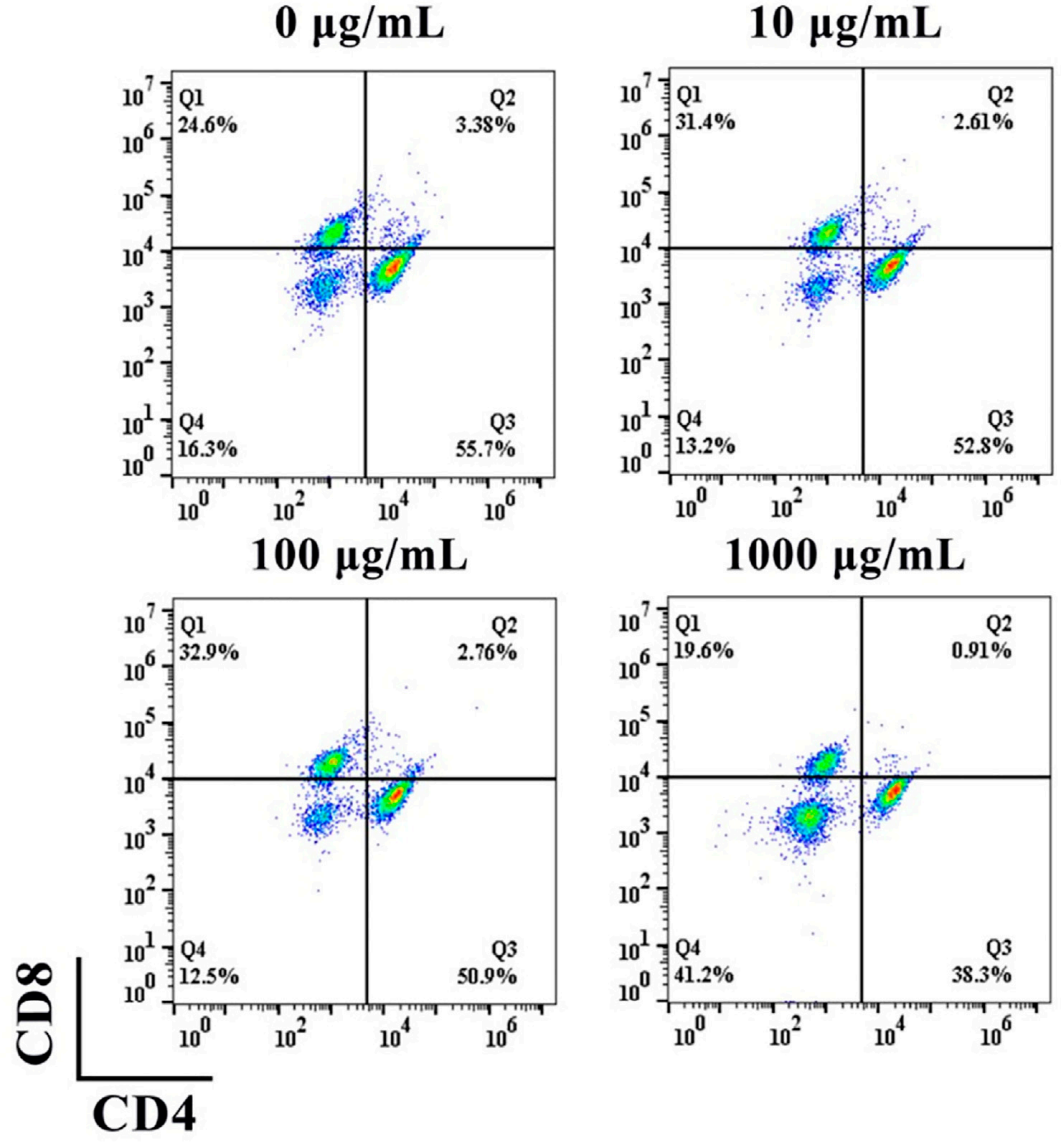
Flow cytometry analysis of the effect of nHA on T cell differentiation.

To evaluate the effect of nHA on the differentiation of T cells to CD4^+^ or CD8^+^ T cells, we used different concentrations of nHA to co-culture with T cells for 5 days. As indicated in Figure 4, in the presence of 100 μg/ml nHA, the proportion of differentiated T cells was more than those of the other groups. In each group, the proportion of CD4^+^ T cells was greater than that of CD8^+^ T cells. Interestingly, the 1,000 μg/ml group had the most undifferentiated and normal living cells than other groups. This may be due to the effect of feedback-regulated balance of growth and differentiation (Bocharov et al., 2011). We found that 100 μg/ml of nHA had a large effect on T cell differentiation, with 50.9% CD4^+^ and 32.9% CD8^+^ T cells. This concentration of nHA has the greatest effect on T cell differentiation compared with other groups. The differentiated T cells may produce more various cytokines, which may offer interesting information for the regulation on osteogenic differentiation. Therefore, it is essential to further investigate the effect of this condition on T cells on osteogenic differentiation. Therefore, 100 μg/ml of nHA was selected in subsequent experiments.

#### 3.2.1 MC3T3-E1 Cell Viability and Osteogenic Differentiation

**FIGURE 5:**
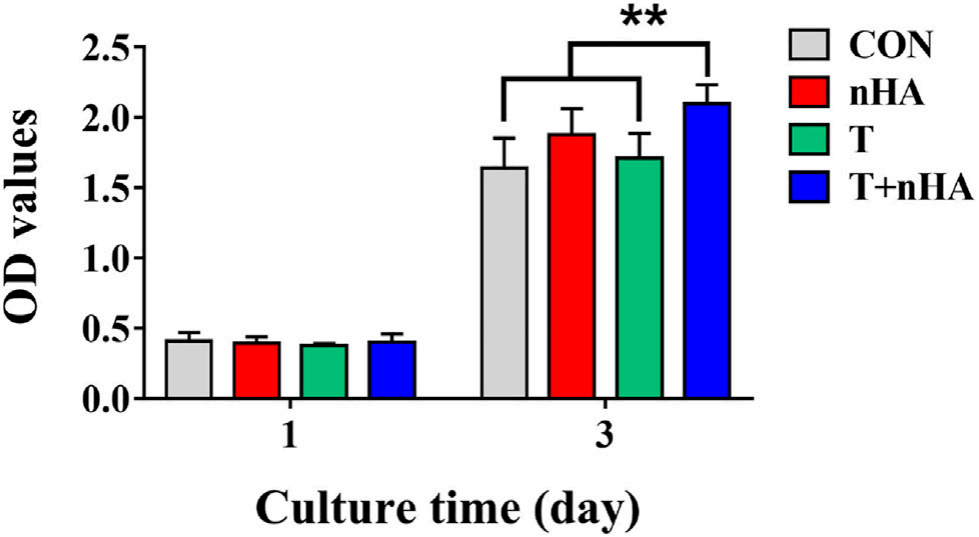
MC3T3-E1 cells viability on various samples for 1 and 3 days. ** represents *p* < 0.01.

The impact of T cell supernatant co-cultured with nHA (T + nHA) on the viability of MC3T3-E1 cells was investigated. After 1 and 3 days of cell growth, the CCK-8 viability test was conducted. With the increase of culture time, the viability of MC3T3-E1 cells was significantly increased. However, after 3 days culture, the supernatant of T + nHA significantly affected the viability of MC3T3-E1 cells, compared with the control and the supernatant of T cells (Figure 5).

**FIGURE 6:**
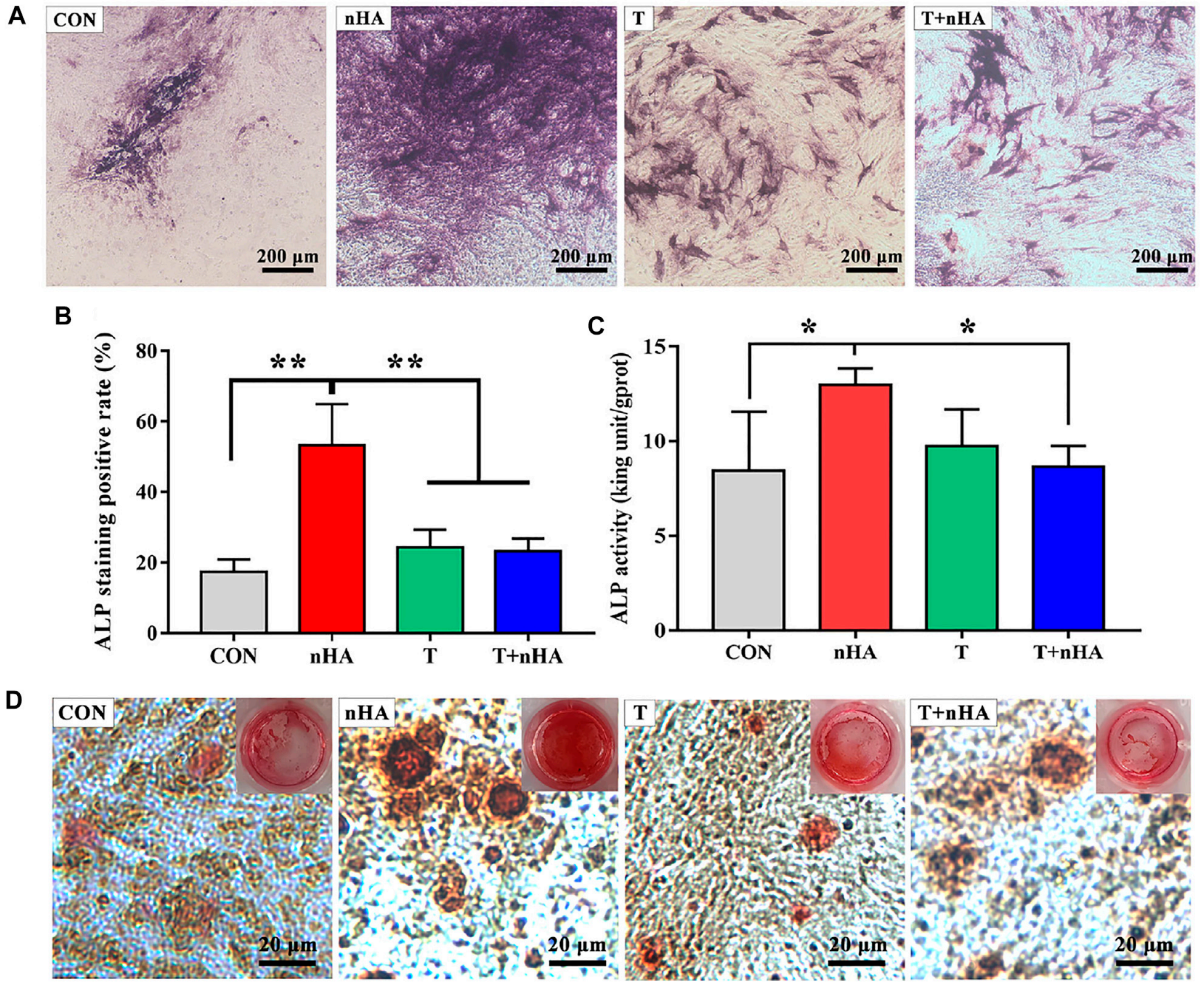
**(A)** ALP staining images. **(B)** The quantitative assessment of the ALP staining results was performed using ImageJ. **(C)** ALP activity of MC3T3-E1 cells on different samples after 14 days of culture. * represents *p* < 0.05 and ** represents *p* < 0.01. **(D)** ARS stained images of MC3T3-E1 cells on different samples after 14 days culture.

ALP is the most significant biological marker of osteogenic differentiation and new bone production (Seo et al., 2010; Zhou et al., 2020; Liu X.-W. et al., 2021). MC3T3-E1 cells were cultured in the supernatant of T + nHA for 14 days and subsequently stained for detecting alkaline phosphatase activity. As shown in Figures 6A,B, it was found that in the group of nHA supernatant, the number of stained cells and the staining area was larger than other groups, and the staining in the group of nHA supernatant is deeper. The other groups had similar staining. Further, the ALP activity of MC3T3-E1 cells was analyzed by an ALP assay kit (Ehara et al., 2003). As depicted in Figure 6C, the supernatant of T + nHA significantly inhibited the ALP expression of MC3T3-E1 cells compared with the supernatant nHA.

In addition, mineralized nodules formed by extracellular matrix Ca deposition were stained with ARS, a dye that binds to Ca ions. By the ARS staining, bright red color of calcified nodules was found in all groups (Figure 6D). However, in the group containing supernatants of T + nHA, the ARS staining showed that the number of calcified nodules was less, and the area of calcified nodules was smaller than in the other groups. The above indicates that the supernatant of T + nHA inhibited the stromal mineralization of MC3T3-E1 cells. Based on the results of ALP and ARS, we found that the supernatant of 100 μg/ml nHA co-cultured with T cells inhibited the osteogenic differentiation of MC3T3-E1 cells. This concentration of needle-like nHA mediated the most differentiation of T lymphocytes into CD4^+^ cells. It may be due to cytokine secretion by total CD4^+^ T cells that inhibited osteogenic differentiation (Shao et al., 2020). And the specific cytokine secretion needs to be further explored in future studies.

## 4 Conclusion

In summary, compared with 0–100 μg/ml nHA, 1,000 μg/ml nHA significantly promoted the growth of T cells and inhibited their apoptosis. In addition, 100 μg/ml nHA was more favorable to cell differentiation into CD4^+^ T cells. Moreover, the supernatant of T cells co-cultured with nHA greatly suppressed the osteogenic differentiation of MC3T3-E1 by reducing the production of ALP and calcium nodule compared with the supernatant of nHA. Therefore, this work offers new insight into the nHA-mediated T cell and osteoblast interactions.

## Data Availability

The raw data supporting the conclusion of this article will be made available by the authors, without undue reservation.
